# Improving the DC Dielectric Properties of XLPE with Appropriate Content of Dicumyl Peroxide for HVDC Cables Insulation

**DOI:** 10.3390/ma15175857

**Published:** 2022-08-25

**Authors:** Muneeb Ahmed, Lisheng Zhong, Fei Li, Nuo Xu, Jinghui Gao

**Affiliations:** State Key Laboratory of Electrical Insulation and Power Equipment, Xi’an Jiaotong University, Xi’an 710049, China

**Keywords:** crosslinked polyethylene, dicumyl peroxide, crosslinking degree, HVDC, resistivity, breakdown strength, space charge

## Abstract

In this research, crosslinked polyethylene (XLPE) is developed with selective content of dicumyl peroxide (DCP), and the influence of microstructural properties and chemical composition on the mechanical and direct current (DC) dielectric properties are investigated. The measurements for the microstructural analysis are taken by gel permeation chromatography (GPC), differential scanning calorimetry (DSC), gel content test and Fourier transform infrared (FTIR). The mechanical properties of XLPE are evaluated by hot-set test. The results of microstructural and chemical composition show that the increase in DCP content increases the crosslinking degree from 74.3% to 81.6%, reduces the crystallinity/lamella thickness from 36.8% to 35.5%/7.6–7.1 nm, reduces the average molecular weight between two crosslinks by 0.01 kg/mol and reduces the oxidation level/carbonyl index. The increase in DCP in XLPE samples decreases the permanent elongation from 2.2% to 0% and elongation rate from 300% to 80% of the cable insulation. The rise in DCP content increases the crosslinking degree due to which the DC resistivity and activation energy is increased. The DC breakdown strength at 30–90 °C is increased due to the increase in crosslinking degree and reduction in carbonyl index/oxidation level. The space charge accumulation is measured at 30 °C under 20–60 kV/mm, resulting in less homo-charges and hetero-charges with the increase in DCP. It is proven that the role of appropriate DCP content is vital in increasing the DC dielectric performance, internal material characteristics and mechanical performance of XLPE.

## 1. Introduction

High voltage direct current (HVDC) transmission technology has received extensive courtesy due to its outstanding performance capability, long-distance transmission and large transmission capacity [1]. The growing demand for power transmission poses new challenges for HVDC cables [2,3,4]. For HVDC cable insulation, the most significant electrical parameters for the evaluation are electrical resistivity, breakdown strength and space charge accumulation. The important mechanical parameters for cable insulation are reliant on the evaluation of elongation rate and permanent elongation [5]. The important material parameters for the microstructure analysis are crystallinity, lamella size, molecular weight and chemical composition [6]. The crosslinked polyethylene (XLPE) cable insulation is comprised of low-density polyethylene (LDPE) and percent content of crosslinking agent mainly dicumyl peroxide (DCP). The DCP is mainly used as a catalyst, polymerization initiator and vulcanizing agent in the chemical laboratories. The significance of using peroxide chemical is that it alters the physical properties of the polymeric insulation material. Mainly, its role is to generate chemical bonds among the linear molecules, producing the interaction among linear molecules. This molecular connection produces a network structure which aids in enhancing the polymeric strength and elasticity of polymeric insulation. Hence, the addition of DCP leads to the change of material properties, which becomes a reason for affecting the mechanical and electrical properties of HVDC cable insulation.

The LDPE based XLPE insulation is suitable for HVDC extruded cables due to its higher electrical resistivity, higher breakdown performance and less space charge distortions than the other materials [7]. Shuchao Wang et al. introduced the effect of crosslinking on the space charge characteristics and found that the crosslinking exhibits the negative charge traps into LDPE and surges the deeper charge trap density [8]. Zhimin Yan et al. explain the cross-linking dependence of electrical breakdown performance and trap distribution of XLPE and found that the electrical breakdown strength is regulated by the trap distribution in XLPE insulation [9]. Yingie Li found that the properties of polymers are determined by their structure and temperature stability can be achieved by modifying the polymeric structure [10]. S. Nilsson et al. studied the structural effects on morphology and thermal properties of XLPE on different crosslinking degrees and found that the dimension and perfection of super-molecular structures fluctuate among two materials [11]. Fan-Bo Meng et al. studied the degassing effect on electrical and physico-chemical properties of crosslinked insulation and found that the crosslinking byproducts are not nearly associated, and it is largely associated to the combined structure of internal molecular chains [12]. Zhipeng Lei et al. found that the graphene coating on XLPE can overwhelm the charge injection from the electrode [13]. Benhong Ouyang et al. presented the microstructural change of XLPE on distribution of space charge during thermo-oxidative aging [14]. However, it was found that the relationship between the appropriate content of DCP for XLPE insulation with its material, mechanical and electrical properties have not been investigated before.

In this research, the appropriate content of DCP was used to prepare the XLPE insulation, and the effect of microstructural change and chemical composition on the DC electrical and mechanical properties were investigated. For the microstructural analysis, the crosslinking degree, lamella size, crystallinity, molecular network density, oxidation level and carbonyl index are examined. For the mechanical analysis, the permanent elongation and elongation rate are identified. For the DC dielectric performance, the electrical breakdown strength, electrical resistivity and space charge accumulation are inspected. It was notably observed that the increase in DCP from 1.4% to 2% increases the microstructural properties of XLPE. This increment in microstructural properties increases the DC dielectric and mechanical performance for the XLPE insulation.

## 2. Materials and Methods

### 2.1. Sample Preparation

The commercial LDPE granules with a density of 0.92 g/cm^3^ (23 °C) were mixed with 0.3% MACKLIN C_22_H_30_O_2_S antioxidant 300 through HAPRO twin screw extruder (Hapu electrical technology limited liability company, Harbin, China). The maintained torque was set in between 20 and 35 N.m. The RPM speed of the HAPRO_TM_ pelletizer was set according to the maintained torque obtained by the HAPRO twin screw extruder. The attained granules were then placed in the oven at 70 °C for about 24 h before adding the series of DCP.

The DCP was taken from Shanghai Aladdin Bio-chemicals. The series of DCP was put in to the antioxidize LDPE granules by soaking at a temperature of 70 °C for 24 h. At last, the samples with 100 × 100 × 0.2 mm^3^ were created by hot-pressing. The pre-press molding was performed at 120 °C and 15 MPa for 5 min, while the crosslinking process was completed at 180 °C and 18 MPa for 10 min. Then, the XLPE samples were cooled at room temperature. Before the experiments, all the XLPE samples were degassed in an oven for 24 h at 70 °C to eliminate the residual crosslinking by-products. For ease, the types of samples prepared in this research are listed in Table 1.

### 2.2. Structural Analysis

The crosslinking degree of XLPE was estimated by gel content test. The specimens were trimmed into a mini cubic shape with a 0.2 mm × 0.5 mm × 0.5 mm dimension and then put in a stainless-steel wire mesh bag. After that, the mesh bag was placed in a xylene solution for 24 h at 110 °C. Later, the specimens were dried till a constant weight at 110 °C. At last, the gel content was estimated as the ratio of final weight to initial weight. This test was repeated twice for better accuracy.

The microscopic presence of the functional groups in XLPE insulation was studied by Nicolet IS10 advanced Fourier transform infrared (FTIR) spectroscopy (Thermo-fisher scientific, Waltham, MA, USA). The attenuated total reflectance (ATR) mode was selected for the FTIR spectroscopy. The spectral resolution of ATR-FTIR was better than 0.4 cm^−1^. For measurement precision, an average of 6 specimens with a 200 ± 10 µm thickness was considered. The wavenumber range for ATR-FTIR was set in between 4000 cm^−1^ and 400 cm^−1^ with a scanning of 32 times.

The differential scanning calorimetry (DSC) was utilized to do the structural analysis of XLPE insulation. In this characterization, the tested specimen and the reference specimen were differentiated in terms of an energy change over temperature difference. The DSC 250 system was used to identify the crystallinity of XLPE. The temperature range was set in between 30 °C and 200 °C with a heating rate of 10 °C/min. The temperature accuracy was ±0.05 °C and temperature precision was ±0.008 °C. The enthalpy precision was ±0.08%. The XLPE samples were heated once to remove the thermal history before the characterization.

To examine the variance in the molecular structure of XLPE insulation, the molecular weight distribution and the molecular weight M_n_ were examined by the gel permeation chromatography (GPC) method. The PL-GPC 220 (Agilent, Santa Clara, CA, USA) was used in this experiment. The flow range of GPC was from 0.1 to 5 mL/min. The flow rate precision was less than 0.02 min. The ambient temperature range was up to 220 °C. Temperature stability was less than 0.05 °C/h. The capacity was up to six 300 × 7.8 mm^2^ columns. The differential refractometer was set as deflection. The Rayleigh scattering angles were 15° and 90°. Firstly, the specimens were liquified in 1,2,4-trichlorobenzene at 150 °C and then they were refined in a 0.5 mm metal net refiner for the removal of undissolved gels and particles in the material. The measurement was taken with a pace of 1.00 mL/min at 150 °C. The calibration type was considered narrow standard, with molecular weights ranging from 4.96 × 10^2^ to 5.34 × 10^6^.

The hot-set test was conducted to identify the permanent elongation and elongation rate of XLPE insulation. The dumbbell-shaped specimens with 1 mm thickness and 75 mm length were prepared for this test. Before the test, the length of 20 mm was marked as *L*_0_. The load of 20 N/cm^2^ was applied and specimens were put in an oven at 200 °C for 15 min, the new length between marks was measured and labeled as *L*_1_. In the end, the attached weight was separated and the specimens were placed in the oven for 5 min before cooling down until 30 °C. Then, the length among the marks *L*_2_ was evaluated again to identify the permanent elongation. The elongation rate λ_1_ = *L*_1_/*L*_0_ and permanent elongation *λ*_2_ = *L*_2_/*L*_0_ were calculated [10].

### 2.3. DC Performance Measurements

The electrical resistivity was investigated by a 3-terminal electrode system containing a sample positioned in an oven. The SL300 ±40 kV power supply (Spellman, New York, NY, USA) and electrometer Keithley 6517B (Tektronix, Beaverton, OR, USA) was utilized in this experiment. The diameter for the HV, measuring and guard ring electrode was 46 mm, 26 mm and 46 mm, respectively. The measurements of conduction current were done at 30–90 °C under 10–60 kV/mm for 30 min. The data acquisition of conduction current was completed on LabView software. The value of conduction current was measured by considering the average of last 60secs. The experiment was repeated for 3 samples to obtain precise measurements. The reciprocal of electrical conductivity was considered to achieve electrical resistivity.

The breakdown strength was measured by putting the sample among two stainless steel electrodes with a diameter of 25 mm. The electrode system was placed in vegetable insulation oil to evade the flashover voltage. This breakdown system was put in an oven to vary the temperature from 30 °C to 90 °C. The direct current (DC) ramp voltage of 1 kV/s was applied until DC breakdown voltage exists. To obtain the DC breakdown strength, the ratio of breakdown voltage and breakdown point thickness was measured.

The pulsed electro-acoustic (PEA) method was used to evaluate the space charge accumulation. The silicon oil was put on the sample as a coupling agent. The 220 µm thick sample was placed among the lower aluminum electrode and upper semiconductive electrode. The electric stress was considered from 20 to 60 kV/mm at room temperature. The measurements were taken for 30 min.

## 3. Results and Discussion

### 3.1. Microstructural Analysis

**Figure 1:**
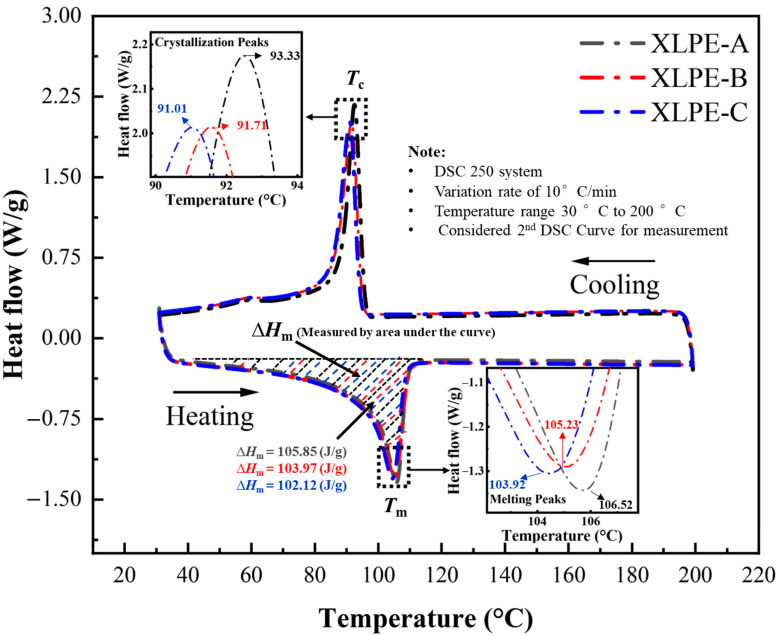
Determination of crystallization parameters of XLPE prepared with DCP content of 1.4% (XLPE-A), 1.7% (XLPE-B) and 2% (XLPE-C). The insets of this figure indicate the melting peaks ($T_{m}$) and crystallization peaks ($T_{c}$) of XLPE-A, XLPE-B and XLPE-C. The melting enthalpy (Δ*H*_m_) is measured by evaluating the area under the curves.

The microstructural properties of samples were identified by the analysis of DSC and gel content test. In Figure 1, the DSC curves of XLPE with appropriate content of DCP are shown. It determines that the crosslink network have a substantial effect on all thermal properties. From the ratio of melting enthalpy of fully crystallized polyethylene and the melting enthalpy of the specimen, the crystallinity *X*_c_ is computed with Equation (1).

(1) $$X_{c}=\frac{\Delta H_{m}}{\Delta H_{0}}\;\times 100$$

where Δ*H*_m_ is the melting enthalpy of the specimen; Δ*H*_0_ is the melting enthalpy of fully crystallized polyethylene, which is about 287.3 J/g.

The lamella thickness *L* of XLPE is calculated by the following Equation (2).

(2) $$L=\frac{{2\sigma }_{e}}{\Delta H_{m0}}\frac{T_{m0}}{T_{m0}{-T}_{m}}$$

where $\sigma_{e}$ denotes the surface free energy per unit area of basal surface, 9.3 × 10^−2^ J/m^2^; $T_{m0}$ indicates the equilibrium melting temperature of the infinitely thick crystal, 414.6 K; $T_{m}$ illustrates the melting temperature of specimen; $\Delta H_{m0}$ is the melting enthalpy of fully crystallized polyethylene per unit volume, 2.88 × 10^8^ J/m^3^.

From Table 2, it can be seen that, from XLPE-A to XLPE-C, the increase of DCP content reduces the melting and crystallization temperature. This decrease in melting temperature decreases the melting enthalpy from 105.85 J/g to 102.12 J/g. This specific decrease in melting enthalpy reduces the crystallinity from 36.84% to 35.54%. This reduction in crystallinity reduces the hardness and density of the material. It is known that the lower crystallinity decreases the thermal conductivity of polymer [15]. As the heat will be transported hardly along XLPE chains than perpendicular to the XLPE chain, XLPE reveals less anisotropy in their thermal conductive properties. The lamella thickness is reduced with the increase in DCP from 7.63 nm to 7.11 nm. A similar decrease in *T*_c_, *X*_c_, and *T*_m_ with an increase in DCP is observed by the author for the composite-based XLPE insulation [16].

The greater lamella size will support the polymer chains to increase the melting temperature, thus reducing the melting enthalpy and crystallinity of the material. The polyethylene (PE) is a semi-crystalline material whose molecular chains are folded to develop lamellar crystals, resulting in a form of spherulites. The increase of crosslinking agent DCP develops a crosslinking network which inhibits the molecular chain movements and spherulite growth, resulting in a decreasing crystallinity. It is clear from the DSC investigation that the crosslinks are not evenly distributed resulting in curve areas with divergent crosslink densities.

**Figure 2:**
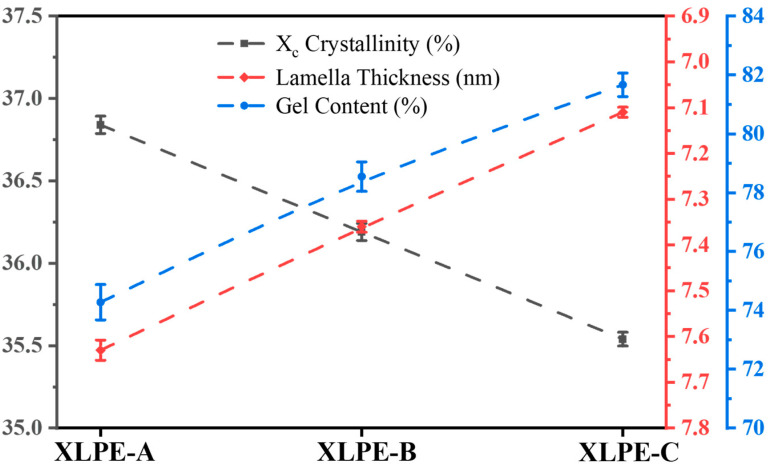
Comparative analysis of crystallinity, lamella thickness and gel content of XLPE prepared with DCP content of 1.4% (XLPE-A), 1.7% (XLPE-B) and 2% (XLPE-C).

In Figure 2, the comparison of crystallinity, lamella thickness and gel content are shown. The gel content is referring as the crosslinking degree of XLPE insulation. It shows that crosslinking degree has an inverse relation with crystallinity and lamella size. Moreover, the increase of DCP from XLPE-A to XLPE-C decreases the crystallinity by 1.3%, decreases the lamella thickness by 0.52 nm, and increases the crosslinking degree from 74.3% to 81.6%. The increase of DCP intensify the microstructural properties of the cable insulation to a specific degree, but with the increase of DCP, the cross-linking by-products are also increased, which affects the electrical properties of the HVDC XLPE cable insulation [17,18].

### 3.2. Molecular Network Density Analysis

**Figure 3:**
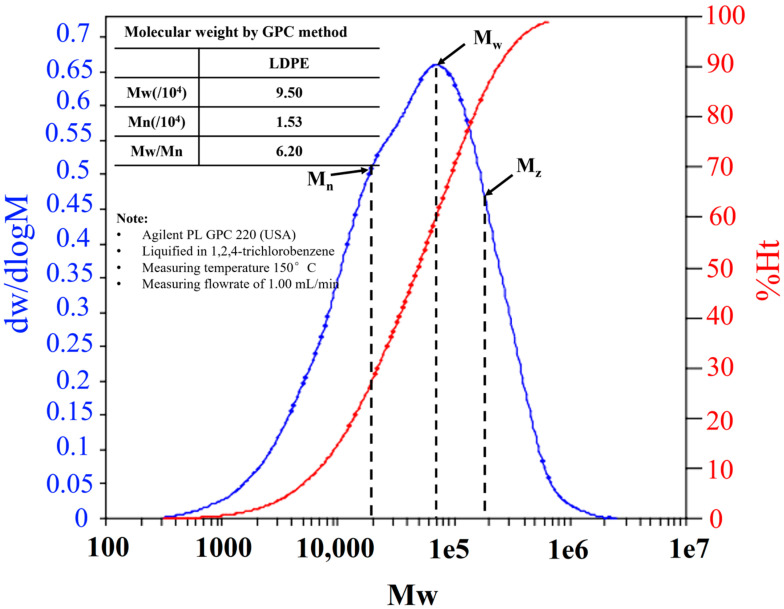
Molecular weight distribution of LDPE for the identification of molecular network density of XLPE by GPC technique.

The molecular network density is established by the hot-set test and GPC. In Figure 3, GPC is used to evaluate the average molecular weight of LDPE. Surely, the samples have the same molecular structure but different crosslinking degrees due to which the crosslinking network density fluctuates. It is found from the gel permeation chromatography that the $M_{n}$, the average molecular weight of LDPE is 1.53 × 10^4^. The $M_{w}$, the weighted average of LDPE is 9.5 × 10^4^. The ratio of $M_{n}{/M}_{w}$ is termed as the polydispersity index and it illustrates the distribution width. The value of the polydispersity index of LDPE is 6.20. The higher polydispersity index illustrates the heterogeneity in crosslinking, network formation, branching and chain length. The $M_{z}$ average is the molecular weight define as the mass of polymer molecule correlated with toughness that is 2.8 × 10^5^ for LDPE.

Therefore, by combining the outcomes of hot-set test and GPC, the average molecular weight among the two crosslinks is evaluated on the bases of the statistical mechanical theory of rubber elasticity, supposing an affine network model [19].

(3) $$M_{C(\mathrm{Hotset})}=\frac{1}{\left(\frac{2}{M_{n}}+\frac{\sigma }{\rho_{200}RT\left(\lambda_{1}-\frac{1}{\lambda_{1}^{2}}\right)}\right)}$$

where the elongation rate *λ*_1_ = *L*_1_/*L*_0_ and corrected stress σ is calculated by hot-set test, ρ_200_ is the polymer density at 200 °C (here 753.6 kg/m^3^), R denotes a gas constant which is equal to 8.3144 J/(mol∙K), *T* indicates the temperature in K (here 473K), *M*_c(Hotset)_ is the average molecular weight between two crosslinks.

**Figure 4:**
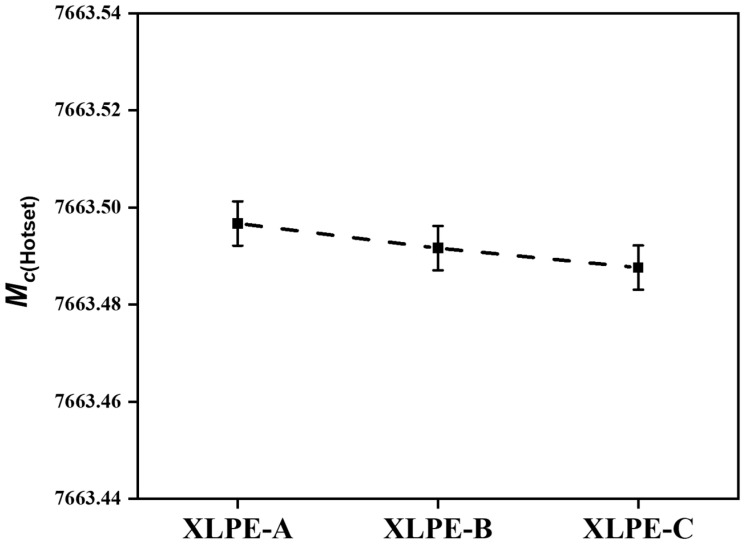
Average molecular weight among two crosslinks for XLPE prepared with DCP content of 1.4% (XLPE-A), 1.7% (XLPE-B) and 2% (XLPE-C).

From Figure 4, it is found that the average molecular weight between the two crosslinks is reduced with the increase of DCP content. Therefore, this minor reduction in the molecular weight among two crosslinks will lessen the strength of chemical resistance. The reduction in molecular weight will help to reduce the damage to the main chains of molecules before affecting the strength of the material. Due to less damage to the main chains of XLPE, the XLPE will result in the strengthening of DC dielectric properties as shown further in this article.

### 3.3. Chemical Composition Analysis

**Figure 5:**
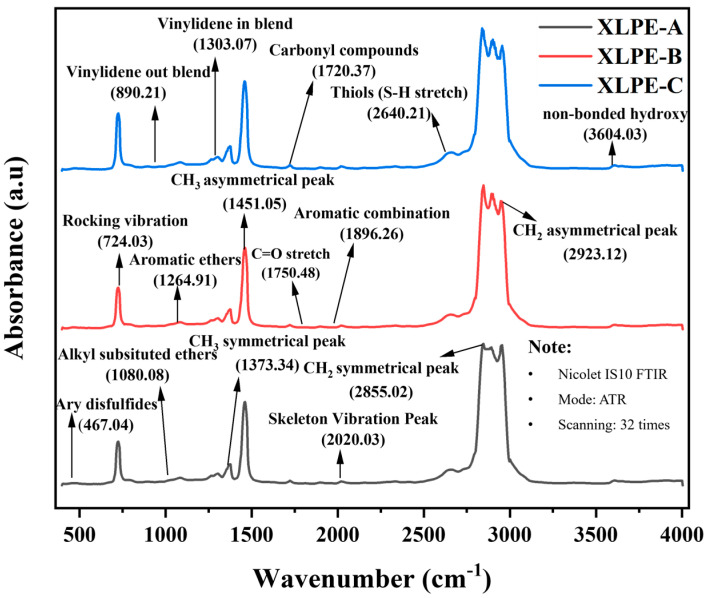
Identification of Functional groups/recognized peaks by Fourier transform infrared (FTIR) for XLPE prepared with DCP content of 1.4% (XLPE-A), 1.7% (XLPE-B) and 2% (XLPE-C).

The ATR-FTIR spectroscopy is used to investigate the chemical composition of XLPE insulation samples. In Figure 5, the FTIR plot for the samples is shown. The absorbance indicates the strength of the bond presence.

The peak mark at 467 cm^−1^ specifies the presence of Aryl disulfides S-S stretch from the thilos and thio-substituted compounds. The peak mark at 2640 cm^−1^ specifies the thiols compounds showing the S-H stretch. The presence of S-S and S-H stretch shows the occurrence of antioxidants in XLPE materials. It is stated that the addition of antioxidants in polymers improves the electrical and material characteristics [20]. The peak mark at 724 cm^−1^ corresponds to the methylene CH_2_ rocking vibration. The peaks at 1373 cm^−1^ and 1451 cm^−1^ specify the presence of CH_3_ symmetrical and asymmetrical bending. The peak at 1720 cm^−1^ shows the carboxylic acids/ketones indicating the carbonyl compounds in the material. The absorbance of important peaks is shown in Table 3.

**Figure 6:**
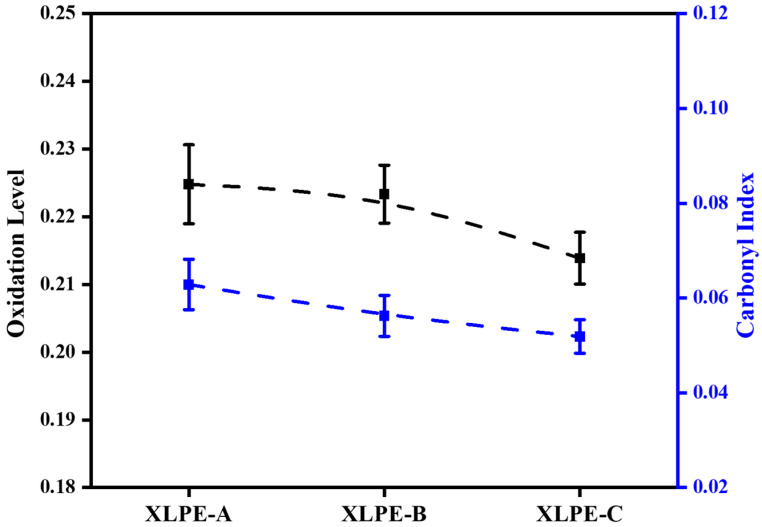
Oxidation level/carbonyl index for XLPE prepared with DCP content of 1.4% (XLPE-A), 1.7% (XLPE-B) and 2% (XLPE-C).

The oxidation level of XLPE is evaluated by the ratio of 1720 cm^−1^ and 1373 cm^−1^. The absorbance ratio of the methylene group and carbonyl group can predict the degradation of insulation [21]. Moreover, the ratio of the 1720 cm^−1^ carbonyl group and 724 cm^−1^ rocking vibration peak can predict the carbonyl index of the XLPE [22]. It is seen from Figure 6 that an increase in DCP content in XLPE samples reduces the oxidation level and carbonyl index. The higher oxidation level and carbonyl index lead to a reduction in mechanical and electrical properties of HVDC cable insulation. During the aging mechanism, the higher carbonyl index/oxidation level may lead to the degradation of cable insulation. From Figure 6, It is clear that the increase in DCP in XLPE will increase the crosslinking ability of polymer and reduce the carbonyl index and oxidation level resulting in strengthened DC dielectric performance and material characteristics.

### 3.4. Mechanical Analysis

**Figure 7:**
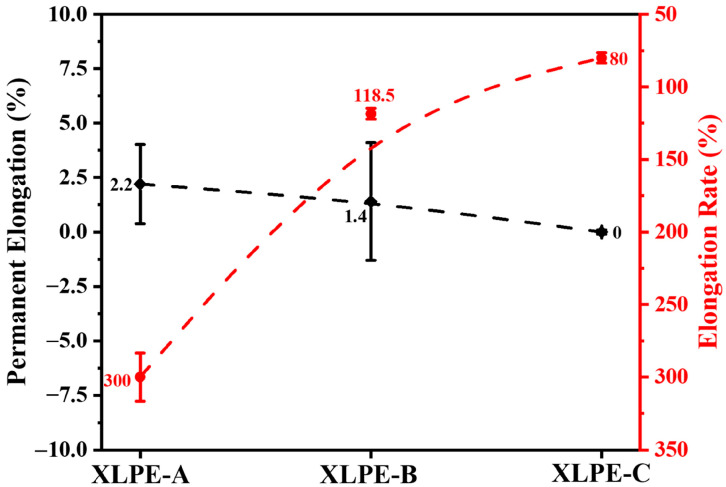
Identification of permanent elongation (%) and elongation rate (%) by hot-set test for XLPE prepared with DCP content of 1.4% (XLPE-A), 1.7% (XLPE-B) and 2% (XLPE-C).

The mechanical properties of XLPE are evaluated by hot-set test. The hot-set test of the cable insulation was measured according to IEC 60811-507:2012. The minimum requirement for the elongation rate should be less than 175% and permanent elongation should be less than 15% for cables insulation. It is worth noting that the variation in the microstructure caused by the increase in DCP content will result in increased mechanical properties for the HVDC crosslinked insulation samples. In Figure 7, the increase in DCP in XLPE samples decreases the permanent elongation from 2.2% to 0% and elongation rate from 300% to 80% of the cable insulation. Therefore, it can be understood that the increase of DCP increases the elongation rate and permanent elongation of XLPE. The increase in DCP in XLPE samples will increase the crosslinking degree which will increase the polymer chains that are interconnected in the network. Therefore, when the cable samples will go under hot-set examination, the higher crosslinking degree will support the sample to attain the precise permanent elongation of less than 15% and elongation rate of less than 175% for HVDC cable insulation. It is found that XLPE-C has the best mechanical performance and it is noted that the DCP content should be greater than 1.4% to pass the cable hot-set test.

### 3.5. Electrical Resistivity and Activation Energy Analysis

**Figure 8:**
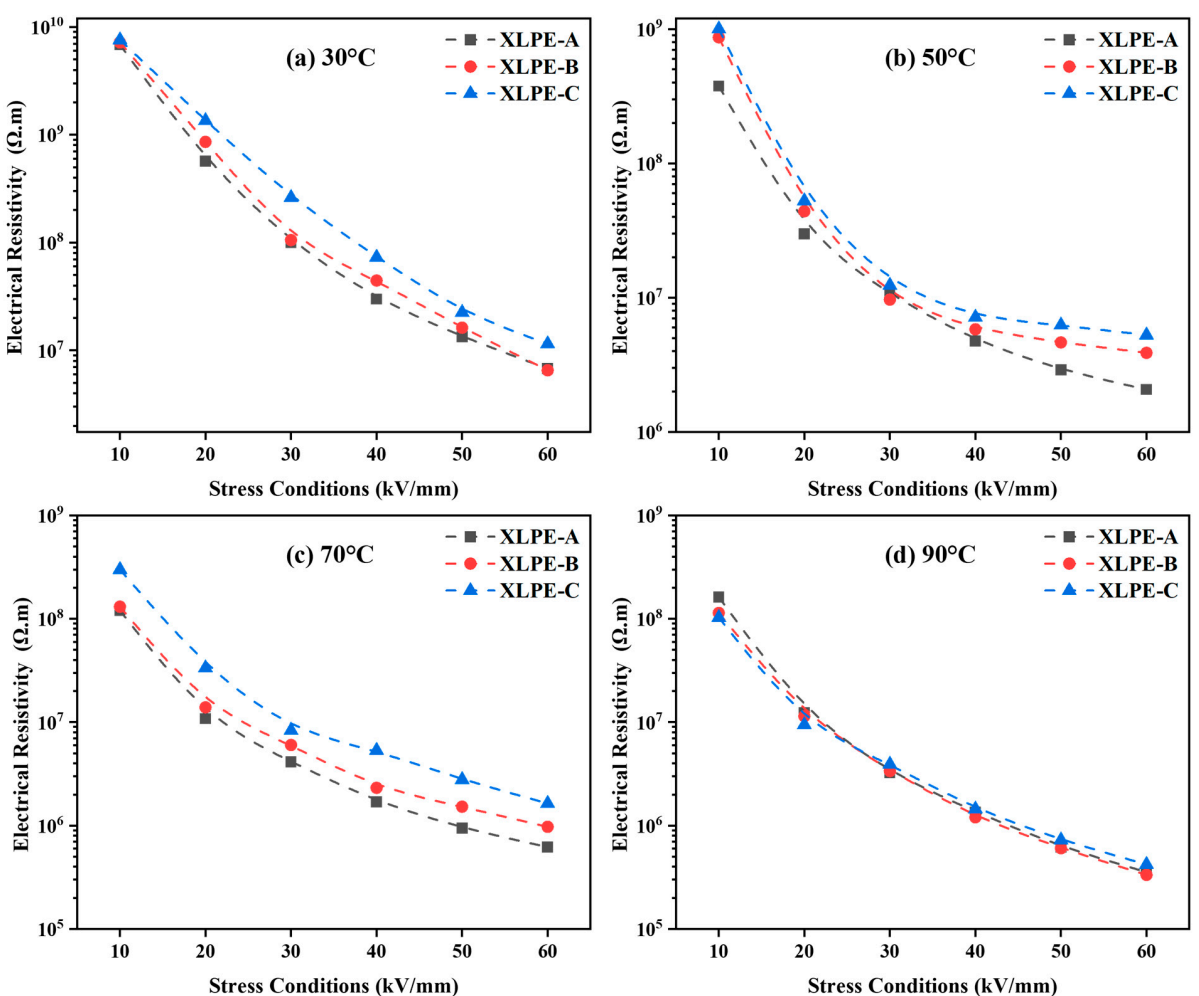
Electrical resistivity (Ω.m) at 30–90°C under 10–60kV/mm stress conditions for XLPE prepared with DCP content of 1.4% (XLPE-A), 1.7% (XLPE-B) and 2% (XLPE-C). (**a**) At no load temperature condition i.e., 30 °C. (**b**) At operating temperature condition i.e., 50 °C. (**c**) At operating temperature condition i.e., 70 °C. (**d**) At full load temperature condition i.e., 90 °C.

The electrical resistivity was measured under 10 kV/mm–60 kV/mm at 30–90 °C. From Figure 8, it is found that an increase in DCP increases the DC resistivity at 30–90 °C for every stress condition. It is seen that temperature has an impact on DC resistivity. The temperature rises from 30 °C to 90 °C diverges the microstructural properties of the material due to which the DC resistivity is reduced. Due to the increase in DCP from XLPE-A to XLPE-C, the reduction of carbonyl index, oxidation level, crystallinity and molecular weight between two crosslinks becomes one of the reasons for the increase of DC resistivity. The crystallinity and electrical resistivity are inversely related. The increasing DCP content from 1.4% to 2% develops an increasing crosslink network which inhibits the molecular chain movements and spherulite growth resulting in a decreasing crystallinity. This reduction in crystallinity increases the DC resistive properties of material. It has been reported that the crosslinking degree and chemical composition has a significant influence on the DC conductance performance of the XLPE [23,24,25]. Referring to Figure 6, the chemical change in the material has also a direct influence on the DC resistive properties. The decreasing trend of carbonyl index and oxidation level increases the DC resistive properties in a similar trend. In Figure 8, with the increase of stress conditions the electrical resistivity is reducing as a trend XLPE-A<XLPE-B<XLPE-C. The increasing trend of stress condition from 10 kV/mm to 60 kV/mm will ease the electrons to transfer from conduction band of crystalline portion to other conduction band of crystalline portion resulting in reducing DC resistivity. Moreover, the significant difference in DC resistivity among XLPE samples is also due to the difference in crosslinking degree. The greater crosslinking degree will increase the crosslinking chains due to which the DC dielectric performance of cable insulation is improved. In Figure 8d, the temperature at 90 °C corresponds to the full load operating temperature of cable insulation, the difference in DC resistivity among the samples is minor because the higher temperature will let the crystal structure warm and molecules will move faster, resulting in lower DC resistivity with less difference among them.

It is known that the conduction process of the samples is in accord with the electron hopping conductance. Therefore, at a low field stress (*E* < 100 kV/mm), the hopping conduction caused by the thermal excitation electrons gets priority, and the related DC conductivity can be represented as shown in Equation (4) [26]:

(4) $$\gamma (T)=\gamma_{0}\exp(-\frac{E_{a}}{RT})$$

where, γ(T) is the DC conductivity as the function of temperature, R illustrates the gas constant, T denotes the temperature in Kelvin, $E_{a}$ is the activation energy in kJ/mol.

The activation energy is calculated from the above Arrhenius Equation (4), by taking the logarithmic function on both sides of the equation, the ln γ−1/*T* is illustrated as a straight line with a slope of *E*_a_/R.

**Figure 9:**
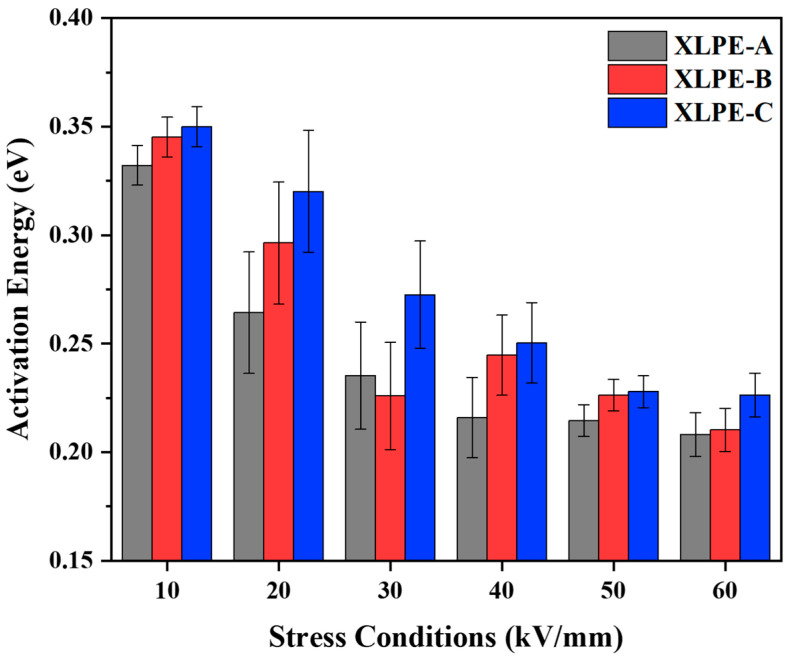
Activation energies (eV) under various stress conditions (kV/mm) of XLPE prepared with DCP content of 1.4% (XLPE-A), 1.7% (XLPE-B) and 2% (XLPE-C).

In Figure 9, it is seen that the activation energy is increased with the increase of DCP content in samples resulting in higher activation for XLPE-C. The main reason for the difference in activation energy is contributed to the structural variation of material. The polymeric insulation is comprised of amorphous and crystalline portions, resulting in a long-range disorderly and short-range orderly structure. It is known that the energy band occurs in the contained crystalline portion, and the electrons are transferred from the conduction band of the crystalline portion to another conduction band of an adjacent local crystalline portion. They must overwhelm a potential barrier degree by means of tunneling or hopping through an amorphous portion. Therefore, the content of DCP plays a vital role as a crosslinking agent which increases the molecular chains and crosslinking process in the material. The results of Figure 8 and Figure 9 illustrate that the rise of DCP content increases the difficulty of electrons overcoming a potential barrier and resulting in higher DC resistivity for the material. From Figure 8, it is noted that the increase in stress conditions will lessen the activation energy of molecules which will fasten the reaction rate for the material. It is noted that under cable operating conditions, the lower activation energy will let the electrons transfer faster from the conduction band to the crystalline region resulting in an early dielectric breakdown of XLPE.

### 3.6. DC Breakdown Analysis

The breakdown strength of XLPE manufactured with appropriate content of DCP is estimated by the Weibull distribution plot [27]. For each of the DCP content, 15 samples are taken to measure the breakdown strength. The experimentally measured breakdown strength for the samples is investigated by using two-parameter Weibull distribution. The confidence interval for the samples is considered 95%. The scale parameter denoted by α illustrates the DC breakdown strength equivalent to a cumulative breakdown probability of 63.2%. The shape parameter denoted by β illustrates the dispersion of breakdown strength. Hence, the greater shape parameter corresponds to the lesser dispersion of breakdown strength. The cumulative breakdown probability of XLPE samples is evaluated at 30 °C to 90 °C temperature conditions. The equation for the two-parameter Weibull distribution is illustrated as:

(5) $$P\left(E\right)=1-\exp{\left(-\frac{E}{\alpha }\right)}^{\beta }$$

where *P(E)* is the cumulative breakdown probability in % and *E* is the applied field strength in kV/mm.

**Figure 10:**
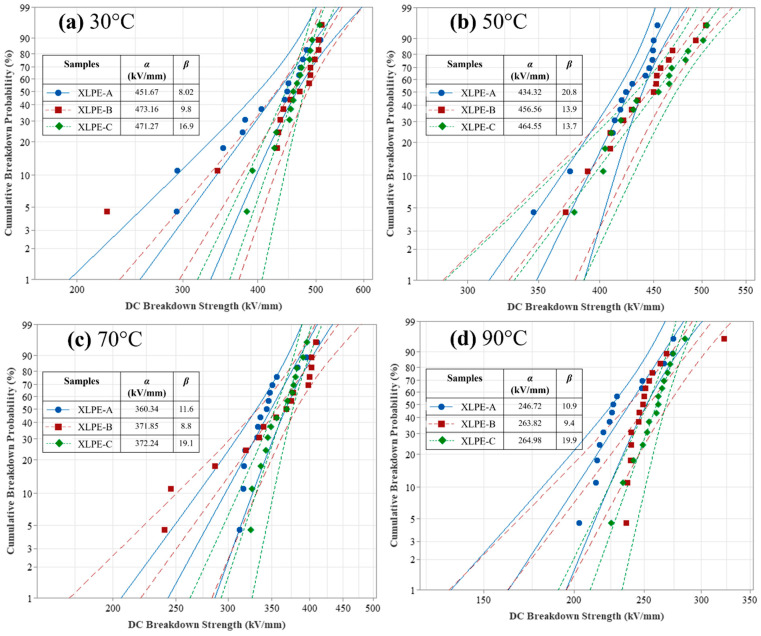
Weibull distribution plot of DC breakdown strength at 30–90 °C for XLPE prepared with DCP content of 1.4% (XLPE-A), 1.7% (XLPE-B) and 2% (XLPE-C). The centered fitted line in the Weibull distribution plot is representing the breakdown probability. The left and the right fitted lines in Weibull distribution plot are representing the 95% lower confidence limit and 95% higher confidence limit of breakdown probability, respectively. (**a**) At no load temperature condition i.e., 30 °C. (**b**) At operating temperature condition i.e., 50 °C. (**c**) At operating temperature condition i.e., 70 °C. (**d**) At full load temperature condition i.e., 90 °C.

From Figure 10, it is seen that the scale parameter *α* is increased with the increase of DCP content within the XLPE samples. It is seen that a higher shape parameter *β* shows less dispersion of DC breakdown strength for the sample XLPE-C. At 30 °C, there is less difference among the DC breakdown strength of XLPE-B and XLPE-C. However, the data dispersion of XLPE-B is higher than XLPE-C. The addition of DCP will increase the microstructural properties and chemical composition resulting in increased DC breakdown strength for XLPE. As stated by researchers, under aging phenomena, the reduction of the carbonyl index and oxidation level helps in the increase of breakdown strength of the material [28]. In this research, the carbonyl index and oxidation level of the material are reduced with the increase of DCP resulting in greater DC breakdown performance for the XLPE-C. From microstructural observation, it is shown that increase in DCP will lower the molecular weight between two crosslinks and increases the molecular chains resulting in less damage to the molecular chain segments and affecting the dielectric properties. Hence, this might be the cause for that, at 30 °C, XLPE-B attains a greater breakdown strength than XLPE-C.

**Figure 11:**
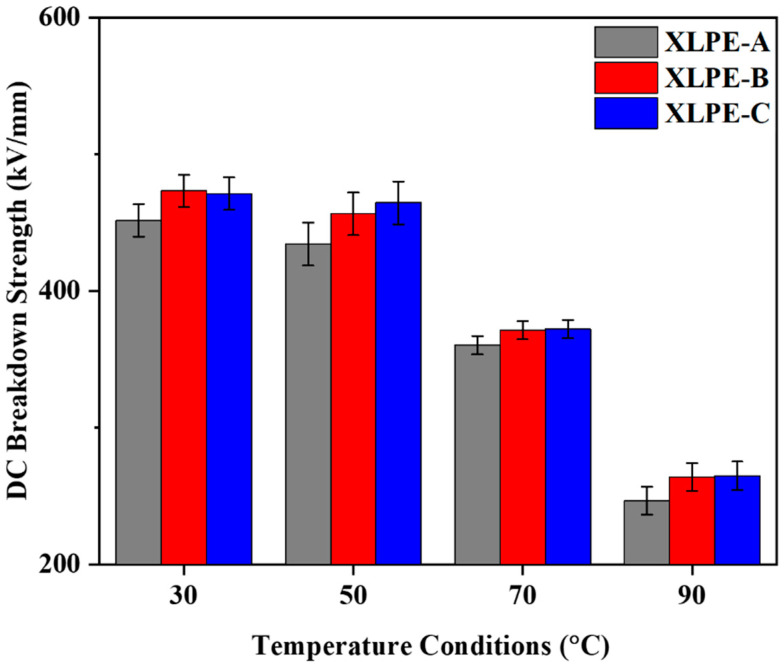
Temperature dependence of DC breakdown strength for XLPE prepared with DCP content of 1.4% (XLPE-A), 1.7% (XLPE-B) and 2% (XLPE-C).

From Figure 11, it is noted that the temperature dependence has a greater impact on the breakdown strength for the XLPE samples manufactured with appropriate content of DCP. It is clear that, due to an increase in temperature, the molecules will move faster, resulting in less DC breakdown strength. In addition, the increase in DCP from XLPE-A to XLPE-C will increase the DC breakdown strength for the temperature conditions. It stated that the influence of crosslinking degree has an impact on the DC breakdown performance of the HVDC cable insulation [9,10,16]. It is clear from the microstructural and DC breakdown strength results that the increase in crosslinking degree with an increase in DCP will increase the DC breakdown performance of the cable insulation.

### 3.7. Space Charge Analysis

**Figure 12:**
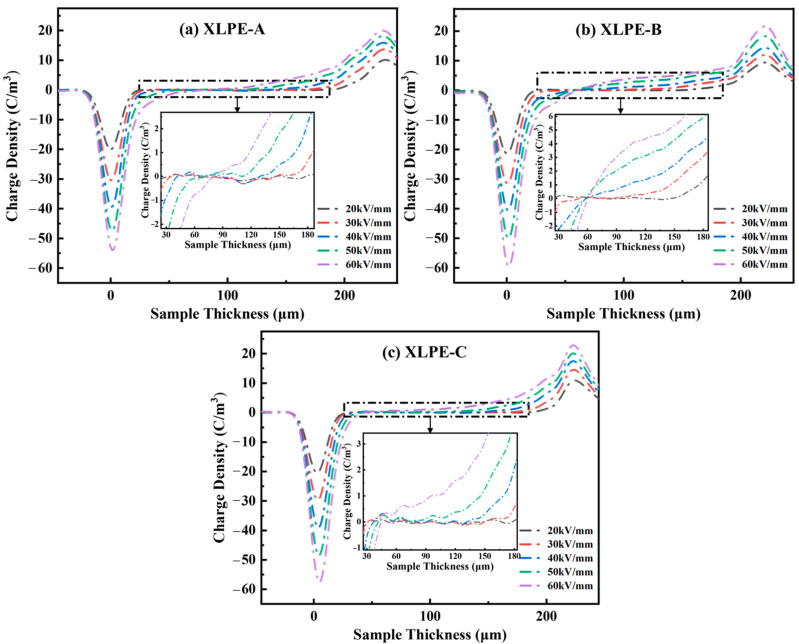
Space charge accumulation at 30 °C under 20–60 kV/mm stress conditions through pulsed-electro acquisition (PEA) technique for XLPE prepared with appropriate content of DCP. The insets shown in this figure illustrates the space charge accumulation between the sample thickness of 30 µm to 180 µm for XLPE prepared with appropriate DCP contents. (**a**) Space charge accumulation for XLPE-A developed with 1.4% DCP. (**b**) Space charge accumulation for XLPE-B developed with 1.7% DCP. (**c**) Space charge accumulation for XLPE-C with 2% DCP.

The space charge accumulation for XLPE insulation manufactured with appropriate content of DCP is measured for 30 min under 20–60 kV/mm at room temperature with the PEA technique. From Figure 12, it is observed that the DCP influences the space charge characteristics. It is seen that the hetero-charge injection in the XLPE is nearly negligible due to the reduction of crosslinking byproducts and internal stress by degassing treatment. Moreover, it is found that the homo-charges on both electrodes are comparably higher for the XLPE-C. It is seen that the increase in crosslinking degree of the samples increases the homo-charges on both electrodes resulting in higher DC breakdown strength for XLPE. From Figure 12a, it is seen that the charge density is negligible and produces minor negative charges under 20–40 kV/mm stress conditions. It is noted that the presence of negative charges within the sample may assist in increasing the field distortion and may help in increasing the breakdown strength for the samples. It is observed from Figure 12, that the increase in stress condition increases the charge density while having negligible negative charges within the sample resulting in a higher homo-charge state near the anode. For Figure 12b, no negative charges are shown for XLPE-B. For Figure 12c, negligible hetero-charges and less homo-charge accumulation are shown within the XLPE-C sample.

## 4. Conclusions

In this research, the XLPE insulation was developed with 1.4–2% DCP content and the influence of microstructural properties and chemical composition on mechanical and electrical properties were investigated. The formation of molecular network density concludes that the reduction in molecular weight between two crosslinks is due to the increase in DCP, lessens the chemical resistance and reduces the damage to the main chains of molecules. The carbonyl index/oxidation level was reduced by the increase of DCP resulting in increase of DC performance of cable insulation. The increase of crosslinking degree due to the increase in DCP strengthened the breaking elongation and elongation rate of cable insulation. With the increase in DCP, the DC breakdown strength and DC resistivity of XLPE were increased due to an increase in crosslinking degree, reduction in carbonyl index/oxidation level, and reduction in crystallinity/lamella size. The homocharge and hetero-charge were reduced due to the increase in crosslinking degree and DCP content. This article concludes that the role of DCP was significant in improving the electrical and mechanical properties of XLPE insulation. The selection of appropriate DCP content of 2% was compulsory to obtain the best performance for HVDC cable insulation.

## Figures and Tables

**Table 1:** Materials prepared.

Designation	LDPE Content/phr	DCP Content/phr	Antioxidant 300/phr
XLPE-A	100	1.4	0.3
XLPE-B	100	1.7	0.3
XLPE-C	100	2.0	0.3

**Table 2:** Parameters of crystallization obtained from DSC curve.

Samples	Δ*H*_m_ (J/g)	$T_{m}({}^{\circ}C)$	$T_{c}({}^{\circ}C)$	*L* (nm)	$X_{c}(\%)$
XLPE-A	105.85	106.5	93.3	7.63	36.84
XLPE-B	103.97	105.2	91.7	7.36	36.19
XLPE-C	102.12	103.9	91.0	7.11	35.54

**Table 3:** Absorbance of identified peaks correspond to the wavenumber (cm^−1^).

Peaks Correspond to the Wavenumber (cm^−1^)	XLPE-A	XLPE-B	XLPE-C
467.04 (Aryl disulfides)	0.111	0.101	0.115
724.03 (Rocking vibration peak)	3.690	3.911	4.510
890.21 (Vinylidene out blend)	0.189	0.173	0.203
1080.08 (Alkyl substituted ether)	0.359	0.322	0.340
1264.91 (Aromatic ethers)	0.483	0.439	0.527
1303.07 (Vinylidene in blend)	0.579	0.554	0.586
1373.34 (CH_3_ symmetrical peak)	1.032	0.985	1.094
1451.05 (CH_3_ asymmetrical peak)	2.826	2.764	3.190
1720.37 (Carbonyl compounds) 1750.48 (C=O stretch)	0.232 0.112	0.220 0.110	0.234 0.124
1896.26 (Aromatic combination) 2020.03 (Skeleton vibration peak) 2640.21 (Thiols S-H stretch)	0.145 0.193 0.744	0.138 0.185 0.706	0.146 0.194 0.740
2855.02 (CH_2_ symmetrical peak)	6.064	6.120	6.184
2923.12 (CH_2_ asymmetrical peak)	5.526	5.635	6.000
3604.03 (Non-bonded hydroxy)	0.264	0.249	0.261

## Data Availability

The data presented in this research are available on request from the corresponding author. The data are not publicly available due to privacy reasons.
